# *Bacillus velezensis* DSM 33864 reduces *Clostridioides difficile* colonization without disturbing commensal gut microbiota composition

**DOI:** 10.1038/s41598-023-42128-8

**Published:** 2023-09-11

**Authors:** Ida Søgaard Larsen, Megan Chenaux, Fergus W. J. Collins, Ana Mandic, Lea B. S. Hansen, Caroline A. S. Lauridsen, Rune F. Haller, Signe Elvig-Jørgensen, Ed Horwell, Jeanett Christiansen, Ana Silva, Maria J. G. T. Vehreschild, Simon M. Cutting, Michael Roggenbuck-Wedemeyer, Nanna Ny Kristensen

**Affiliations:** 1 Novozymes A/S, Bagsværd, Denmark; 2 Novozymes Inc, Davis, CA USA; 3 PrecisionsBiotics Group, Cork, Ireland; 4 Novozymes GmbH, Berlin, Germany; 5 Bioscience Innovation Centre, Sporegen Ltd., 2 Royal College Street, London, NW1 0NH UK; 6 Department of Internal Medicine, Infectious Diseases, University Hospital Frankfurt, Goethe University Frankfurt, Frankfurt am Main, Germany; 7 Department of Biological Sciences, Royal Holloway University of London, Egham, Surrey UK

**Keywords:** Pathogens, Microbiota

## Abstract

Up to 25% of the US population harbor *Clostridioides difficile* in the gut. Following antibiotic disruption of the gut microbiota, *C. difficile* can act as an opportunistic pathogen and induce potentially lethal infections. Consequently, reducing the colonization of *C. difficile* in at-risk populations is warranted, prompting us to identify and characterize a probiotic candidate specifically targeting *C. difficile* colonization. We identified *Bacillus velezensis* DSM 33864 as a promising strain to reduce *C. difficile* levels in vitro. We further investigated the effects of *B. velezensis* DSM 33864 in an assay including human fecal medium and in healthy or clindamycin-treated mouse models of *C. difficile* colonization. The addition of *B. velezensis* DSM 33864 to human fecal samples was shown to reduce the colonization of *C. difficile *in vitro. This was supported in vivo where orally administered *B. velezensis* DSM 33864 spores reduced *C. difficile* levels in clindamycin-treated mice. The commensal microbiota composition or post-antibiotic reconstitution was not impacted by *B. velezensis* DSM 33864 in human fecal samples, short-, or long-term administration in mice. In conclusion, oral administration of *B. velezensis* DSM 33864 specifically reduced *C. difficile* colonization in vitro and in vivo without adversely impacting the commensal gut microbiota composition.

## Introduction

*Clostridioides difficile* is a Gram-positive, anaerobic, spore-forming and toxin-producing bacterium, which can be found in the gastrointestinal (GI) tract of humans and animals as well as being widely present in environmental reservoirs^1^. *C. difficile* is a leading cause of nosocomial antibiotic-associated diarrhoea, and while the species has been found to reside asymptomatically in the GI tract of healthy individuals, asymptomatic colonization can itself be a risk factor for the development of *C. difficile* infection (CDI)^2^.

The human gut microbiota provides an important barrier to *C. difficile* colonization through a number of mechanisms, including limiting the availability of nutrients and germinants^3,4^. Disruption and compositional changes in the gut microbiota, for example through the use of antibiotics, can open a niche for the outgrowth of *C. difficile*^5^. *C. difficile* has the capacity to adapt its metabolism to utilize a variety of different growth substrates made more readily available in the gut as antibiotics reduce the number of microbes which previously would have outcompeted the species for these resources^6^. *C. difficile* also has the potential to interact symbiotically with other opportunistic pathogens taking advantage of a disrupted microbiota, as seen with the interaction between the species and antibiotic-resistant enterococci. Here, as the enterococci expand in response to antibiotic treatment, they can increase the fitness and pathogenicity of *C. difficile* through a process of cross-feeding and nutrient restriction^7^.

As *C. difficile* grows into the niches made available through the disruption of the gut microbiome, the increase in cell numbers along with an inherent depletion of available nutrients can induce the production of *C. difficile*-associated toxins^8,9^. *C. difficile* is known to produce two primary exotoxins, toxin A (TcdA) and toxin B (TcdB), with some strains having been shown to also produce a third binary toxin^10^. These toxins target host colonic epithelial cells, disrupting cytoskeletal structure and tight junctions, leading to apoptosis and a loss of host intestinal barrier integrity^10^. The inflammation in the gut that can be induced by these toxins can lead to the release of additional nutrients which support further growth of *C. difficile,* whilst also restricting the growth of other potential competing bacteria^8^. Clinical manifestations of *C. difficile* toxin production range from mild diarrhea to fulminant colitis^11^.

The recovery of the human gut microbiota after antibiotic treatment plays an important role in reducing the opportunity for the development and progression of CDI^12^. This has led to the development of several therapies aimed at accelerating the restoration of the gut microbiota after antibiotic treatment to a state that can again provide a barrier against *C. difficile* colonization. Fecal microbiota transplant (FMT), whereby healthy donor fecal preparations are used to help reconstitute the diversity and composition of the host microbiome after antibiotic treatment, has proven effective in treatment of recurrent CDI^13,14^. Probiotic supplementation has also been shown to potentially play a role in helping to reduce the incidence of CDI in individuals^15^. In these instances, as the reestablishment of the natural barrier to *C. difficile* colonization provided by the gut microbiota is key in the prevention of post-antibiotic CDI, it is important that the recovery of the microbiota should not be delayed or adversely impacted.

Here, we describe *B. velezensis* DSM 33864, a bacterial strain capable of reducing *C. difficile* loads in vitro and in vivo. *B. velezensis* DSM 33864 was initially isolated from feces of a healthy human donor and showed an increased ability to reduce the growth of *C. difficile *in vitro compared to multiple related members of the *Bacillus* genus^16^. *B. velezensis* is abundant in the traditional Korean food kimchi and has recently gained qualified presumption of safety (QPS) status for food and feed production by the European Food Safety Authority (EFSA)^17,18^. *Bacillus* spores can also be advantageous as they are generally resistant to stomach- and bile acids^19^.

Here we show that *B. velezensis* DSM 33864 was able to reduce *C. difficile* load without impacting commensal microbes or the reconstitution of commensal gut microbiota in healthy or antibiotic-treated mice. This indicates the strain could work alongside the natural barrier to *C. difficile* colonization provided by the host microbiome. Therefore, our results suggest that supplementation of *B. velezensis* DSM 33864 in humans could play a role in reducing the *C. difficile* load in the gut without adversely interfering with the recovery of the microbiota following antibiotic treatment.

## Results

### *Bacillus velezensis *DSM 33864 reduced *C. difficile* levels in vitro

**Figure 1:**
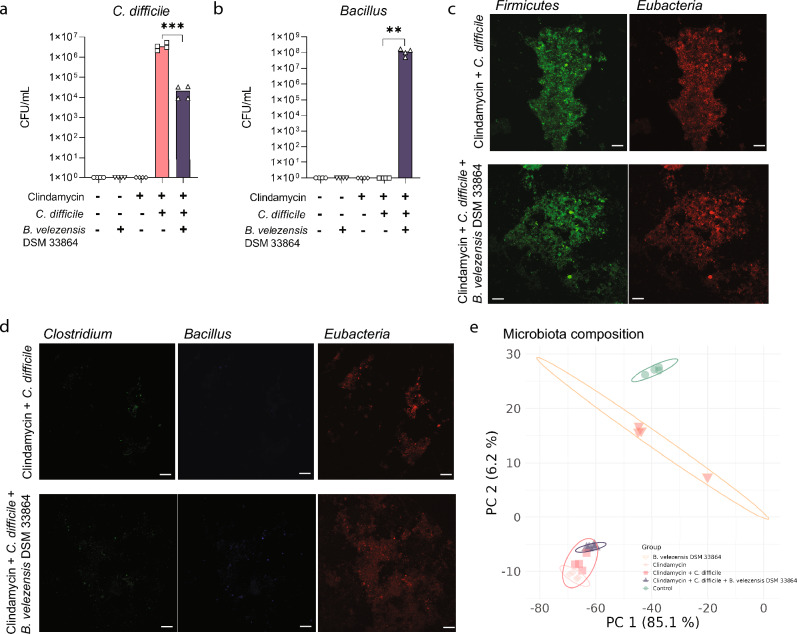
In vitro reduction of *C. difficile* by *B. velezensis* DSM 33864 (**a**) *C. difficile* counts (CFU/mL) from human fecal incubation for 6 h with/without clindamycin, *C. difficile,* or *B. velezensis* DSM 33864 as indicated. (**b**) *Bacillus* counts (CFU/mL) from human fecal incubation for 6 h with/without clindamycin, *C. difficile,* or *B. velezensis* DSM 33864 as indicated. (**c**) Fluorescence in situ hybridization (FISH) of Firmicutes (Green) and Eubacteria (Red) in human fecal incubation for 6 h with clindamycin, *C. difficile* with/without *B. velezensis* DSM 33864*.* (**d**) FISH of *Clostridia* genus (green), *Bacillus* genus (Blue), and Eubacteria (red) in human fecal incubation for 6 h with clindamycin, *C. difficile* with/without *B. velezensis* DSM 33864*.* **e)** Principal component analysis (PCA) of 16S rRNA gene amplicon sequences of human fecal incubation for 6 h with/without clindamycin, *C. difficile,* or *B. velezensis* DSM 33864. (**a, b**) Asterisks indicate *p*-values < 0.05 comparing the indicated groups by one-way ANOVA with multiple comparisons by Tukey’s post hoc.

We tested the ability of *B. velezensis* DSM 33864 to reduce *C.* *difficile* levels in an in vitro human fecal fermentation to mimic a complex environment such as the gut. Here, pooled human fecal samples from healthy donors were treated with or without clindamycin, *C. difficile,* and/or *B. velezensis* DSM 33864 and samples were collected after 0.5 and 6 h of incubation (Fig S1a). In fecal samples incubated with clindamycin and *C. difficile* for 6 h, the *C. difficile* load was significantly reduced by the addition of *B. velezensis* DSM 33864 (Fig. 1a). *Bacillus* viable cell counts increased only where *B. velezensis* DSM 33864 was added to clindamycin-treated fecal samples (Fig. 1b).

Fluorescence in situ hybridization (FISH) of the fecal samples containing clindamycin and *C. difficile* ± *B. velezensis* DSM 33864 revealed that most bacteria in the samples were classified as *Firmicutes* (Fig. 1c, S1b). While *B. velezensis* DSM 33864 reduced *C. difficile* in the fecal samples containing clindamycin and *C. difficile* by 2-logs (Fig. 1a)*,* staining of all fecal *Clostridia* indicated the strain did not affect the overall *Clostridia* presence (Fig. 1d). This was supported by 16S rRNA gene amplicon sequencing showing no significant change in the relative abundance of commensal *Clostridia* (*i.e.*, *Clostridia* not mapping to *C. difficile*) by *B. velezensis* DSM 33864 (Fig S1c). The fecal microbiota composition was statistically similar in all conditions shortly after the addition of clindamycin, *C. difficile,* and/or *B. velezensis* DSM 33864 (PERMANOVA *p* = 0.35, Fig S1d). Addition of *B. velezensis* DSM 33864 significantly increased the Shannon diversity index at 0.5 h (Fig S1e), which was explained by an increase in *Bacillus* abundance (Fig S2). As expected, clindamycin treatment significantly altered the fecal microbiota composition after 6 h of incubation (PERMANOVA *p* = 0.02) as did addition of *C. difficile* (PERMANOVA *p* = 0.03), which was not modified further by adding *B. velezensis* DSM 33864 (PERMANOVA *p* = 0.35, Fig. 1e).

### *B. velezensis *DSM 33864 reduced *C. difficile* colonization in mice

**Figure 2:**
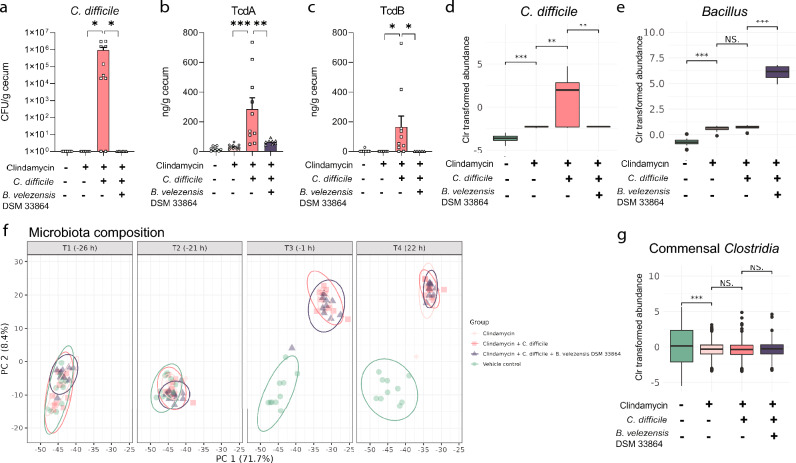
*B. velezensis* DSM 33864 reduced *C. difficile* in a murine colonization model. (**a**) *C. difficile* counts (CFU/mL) from mouse cecum content with/without clindamycin, *C. difficile,* or *B. velezensis* DSM 33864 24 h after oral administration of *C. difficile* to the indicated groups. (**b**) *C. difficile* toxin (Tcd) A levels in mouse cecum content with/without clindamycin, *C. difficile,* or *B. velezensis* DSM 33864 24 h after oral administration of *C. difficile*. (**c**) TcdB levels in mouse cecum content with/without clindamycin, *C. difficile,* or *B. velezensis* DSM 33864 24 h after oral administration of *C. difficile*. (**d**) Center log-ratio (Clr) transformed abundance of *C. difficile* ASV per group assessed by 16S rRNA gene amplicon sequencing of mouse fecal samples 24 h after oral administration of *C. difficile*. (**e**) Clr transformed abundance of ASVs mapping to *Bacillus* genus per group assessed by 16S rRNA gene amplicon sequencing of mouse fecal samples at the same timepoint as in d. (**f**) PCA of 16S rRNA gene amplicon sequences of mouse fecal samples 24 h after oral administration of *C. difficile*. (**g**) Clr transformed aggregated commensal *Clostridia* abundance in the mouse fecal samples 24 h after oral administration of *C. difficile* in the indicated groups. (**a–c**) Asterisks indicate *p*-values < 0.05 comparing the indicated groups by one-way ANOVA with multiple comparisons by Tukey’s post hoc. (**d, e, g**) Asterisks indicate *p*-values < 0.05 and ns indicate *p*-values > 0.05 comparing the indicated groups by t-test.

After demonstrating that *B. velezensis* DSM 33864 reduced *C. difficile* loads in vitro, we investigated the effects in vivo*.* Here, we used a model where mice were colonized by *C. difficile* 24 h after their commensal gut microbiota was disrupted with clindamycin. One group of mice additionally received *B. velezensis* DSM 33864 by oral gavage before and after *C. difficile* administration (Fig S3a). *C. difficile* was measurable in 80% of the ceca from mice which were pre-treated with clindamycin but did not receive *B. velezensis* DSM 33864, which was associated with increased toxin levels (Fig. 2a–c). The levels of both *C. difficile* and the toxins produced by the strain were significantly reduced in the cecum of clindamycin-treated mice in response to oral gavage of *B. velezensis* DSM 33864 (Fig. 2a–c). Microbiota profiling of fecal pellets sampled throughout the study supported the reduction of *C. difficile* by *B. velezensis* DSM 33864 in clindamycin-treated mice (Fig. 2d). This was associated with increased relative *Bacillus* abundance in the mice given *B. velezensis* DSM 33864 (Fig. 2e).

The overall gut microbiota composition was similar between the groups at the start of the study, after which clindamycin drastically shifted the microbiota composition from T3 (− 1 h) (PERMANOVA *p* = 0.002, Fig. 2f) and reduced the Shannon diversity index (Fig S3b). Neither *C. difficile* nor *B. velezensis* DSM 33864 impacted the clindamycin-disrupted fecal microbiota composition in the mice (Fig. 2f). Furthermore, *B. velezensis* DSM 33864 did not change the abundance of specific bacterial genera besides an expected increase in *Bacillus* and reduced *C. difficile* abundance (Fig. 2d,e, S3c). Cyclic lipopeptides were found to be increased by *B. velezensis* DSM 33864 in the colon content of clindamycin- and *C. difficile-*administered mice (Fig S3d), therefore, to further investigate the effects of introducing *B. velezensis* DSM 33864 on the commensal gut microbiota composition, we assessed ASVs classified as *Clostridia* (class). Of the 190 *Clostridia* in the data set, only *C. difficile* was statistically significantly different between the groups. Commensal *Clostridia* were affected by clindamycin, but not by *C. difficile* or *B. velezensis* DSM 33864 in clindamycin-administered mice (Fig. 2g).

### Commensal gut microbes were not affected by *B. velezensis* DSM 33864 in healthy mice

**Figure 3:**
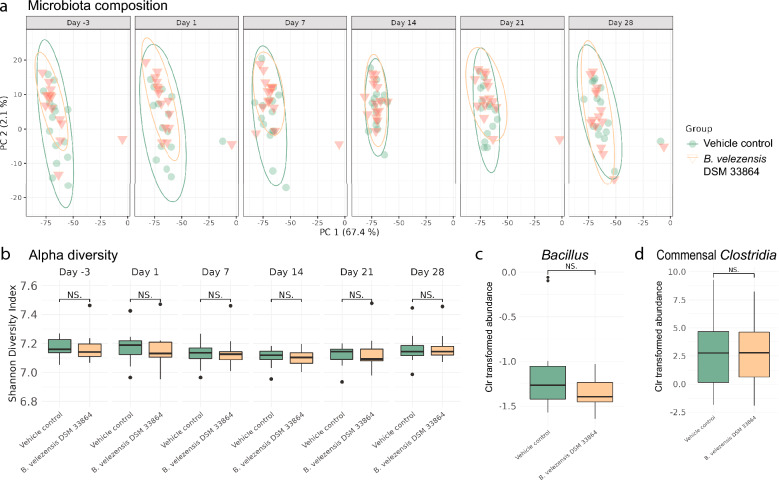
Healthy gut microbiota composition in mice was not affected by *B. velezensis* DSM 33864. (**a**) PCA of 16S rRNA gene amplicon sequences of mouse fecal samples at indicated timepoints during the study in mice prior to (Day -3) and during orally administered vehicle or *B. velezensis* DSM 33864 daily for 14 days (Days 1–14) followed by 14 days washout (Days 21–28). (**b**) Shannon diversity index of fecal samples from the indicated time points during the study. (**c)** Clr transformed relative abundance of *Bacillus* genus per group assessed by 16S rRNA gene amplicon sequencing of mouse fecal samples after 14 days of daily, oral administration of vehicle or *B.* velezensis DSM 33864 (Day 14). (**d)** Clr transformed relative abundance of commensal *Clostridia* abundance in mouse fecal samples after 14 days of daily, oral administration of vehicle or *B.* velezensis DSM 33864 (Day 14)*.* (**b**–**d)** Asterisks indicate *p*-values < 0.05 and ns indicate *p*-values > 0.05 comparing the groups by t-test.

To further investigate potential effects of *B. velezensis* DSM 33864 on the commensal gut microbiota, we conducted a study with healthy, non-antibiotic-treated mice. Potential microbiota compositional changes connferred by *B*. *velezensis* DSM 33864 were monitored during 14 days of daily, oral gavage followed by a 14 day wash-out period (Fig S4a). All mice in the study had a similar baseline microbiota composition (Day -3), which remained stable during the study in both the vehicle-control group as well as the group given *B. velezensis* DSM 33864 (Days 1–28, Fig. 3a). The Shannon diversity index did not differ between the groups over time regardless of whether the microbiota was sampled during the 14 days of daily vehicle or *B. velezensis* DSM 33864 gavage (Days 1–14) or during the following 14-days of wash-out (Days 21–28, Fig. 3b). In mice with a healthy microbiome, the *B. velezensis* DSM 33864 did not increase the relative *Bacillus* abundance (Day 14, Fig. 3c). As with the short-term mouse study (Fig. 2g), *B. velezensis* DSM 33864 did not impact commensal *Clostridia* abundance (Day 14, Fig. 3d).

Additionally, we conducted a 28-day repeat dose safety study in mice with daily oral administration of *B. velezensis* DSM 33864 (Fig S4b). Mice receiving *B. velezensis* DSM 33864 daily showed no treatment-related clinical signs or differences in feed intake or body weight compared to the concurrent control group (Fig S4c,d). Further assessment of selected hematological, clinical chemistry, bone marrow smears, macroscopic and microscopic examinations of selected organs and tissue did not show any adverse effects compared to the concurrent control group. The 28-day oral gavage with *B. velezensis* DSM 33864 established the no-observed-adverse-effect level (NOAEL) in the study to be 1 × 10^8^ CFU/animal/day. Collectively, these studies suggest that daily oral *B. velezensis* DSM 33864 administration did not impact the gut microbiota or cause any adverse effects in healthy, non-antibiotic-treated mice without *C. difficile*-challenge.

### Post-antibiotic reconstitution of the commensal microbiota was not affected by *B. velezensis* DSM 33864

**Figure 4:**
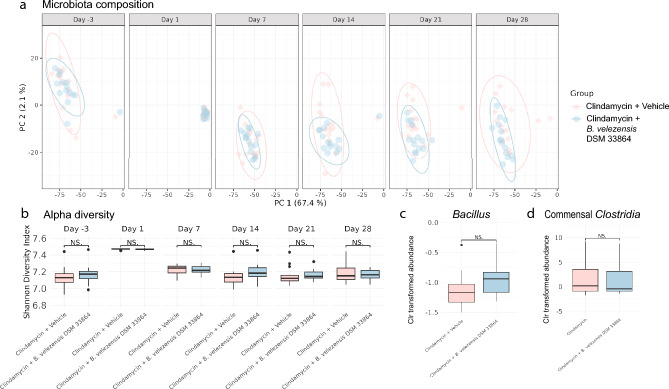
*B. velezensis* DSM 33864 did not impact reconstitution of commensal microbes post-antibiotics. (**a**) PCA of 16S rRNA gene amplicon sequences of mouse fecal samples at indicated timepoints during the study in mice prior to (Day -3) and during orally administered clindamycin followed by vehicle or *B. velezensis* DSM 33864 daily for 14 days (Days 1–14) followed by 14 days washout (Days 21–28). (**b**) Shannon diversity index of fecal samples from the indicated time points during the study. (**c**) Clr transformed relative abundance of *Bacillus* genus per group assessed by 16S rRNA gene amplicon sequencing of mouse fecal samples after 14 days of daily, oral administration of vehicle or *B.* velezensis DSM 33864 (Day 14)*.* (**d**) Clr transformed relative abundance of commensal *Clostridia* abundance in mouse fecal samples after 14 days of daily, oral administration of vehicle or *B.* velezensis DSM 33864 (Day 14). (**b**–**d**) Asterisks indicate *p*-values < 0.05 and ns indicate *p*-values > 0.05 comparing the groups by t-test.

The gradual recovery of the commensal gut microbiota following antibiotic treatment may be delayed by some probiotics^20^. We investigated whether *B. velezensis* DSM 33864 impacted the reconstitution of the commensal gut microbiota composition in a model where mice received a single-dose of clindamycin prior to daily oral gavage of *B. velezensis* DSM 33864 for 14 days, followed by a 14-day wash-out period (Fig S4e). The baseline fecal microbiota composition was similar between groups prior to treatment (Day -3, Fig. 4a). Clindamycin induced a drastic shift in the overall fecal microbiota composition and impacted the Shannon diversity index in samples obtained the day after clindamycin treatment (Day 1, Fig. 4a-b).

Daily oral gavage with *B. velezensis* DSM 33864 did not impact the effect of clindamycin on the fecal microbiota composition. This was observed by a similar response to clindamycin in the groups additionally receiving daily *B. velezensis* DSM 33864 following the single clindamycin dose (Day 1–28, Fig. 4a-b). Over time, the gut microbiota composition reconstituted at the same rate as the vehicle-treated group, thus showing no delay in the natural return of the microbiota composition or diversity (1–28 day, Fig. 4a,b). The relative abundance of *Bacillus* or commensal *Clostridia* were not affected in this study after daily oral *B. velezensis* DSM 33864 administration for 14 days following clindamycin-disruption of the microbiota composition (Day 14, Fig. 4d,e). This indicated that, in addition to *B. velezensis* DSM 33864 not affecting already present commensal *Clostridia,* the strain did not hinder the return and growth of the commensal strains of this bacterial genus*.*

## Discussion

In vitro and in vivo tests have demonstrated the efficacy and specificity of *B. velezensis* DSM 33864 in reducing *C. difficile* load without impacting commensal microbes. The in vitro reduction of *C. difficile* by *B. velezensis* DSM 33864 supports reports of similar activity seen in other strains of *B. velezensis*^21^. The efficacy of *B. velezensis* DSM 33864 in reducing *C. difficile* levels in vitro led us to further investigate the activity and specificity of the strain in more complex in vivo mouse models.

In a short-term mouse model, oral administration of *B. velezensis* DSM 33864 prevented *C. difficile* colonization in mice treated with clindamycin. Interestingly, we found no effect of *B. velezensis* DSM 33864 administration on the remaining gut microbiota, notably commensal *Clostridia* abundances, despite increases in cyclic lipopeptide levels. To further assess the potential impact of *B. velezensis* DSM 33864 on the commensal gut microbiota over a longer time period, a study in healthy mice was conducted. Here, no impact of *B. velezensis* DSM 33864 was found on the gut microbiota of healthy mice after 14 days of daily administration nor after the subsequent 14 days of washout. In addition, daily oral gavage of *B. velezensis* DSM 33864 with a 1 × 10^6^ and 1 × 10^8^ CFU dose for up to 28 days in healthy mice resulted in no adverse effects, confirming *B. velezensis* DSM 33864 is safe to consume.

Clindamycin-challenged mice showed a disrupted gut microbiota composition after approximately 24 h in both mouse models. In the 28-day mouse study, the gut microbiota gradually returned to baseline composition after clindamycin-treatment over the course of the study, largely resembling the features of the pre-antibiotic microbiome after 14 days. *B. velezensis* DSM 33864 did not delay or modify this natural reconstitution of the gut microbiota in the mice, contrasting with the results of Suez et al.^20^.The probiotic consortia tested by Suez et al.^20^ that delayed post-antibiotic microbiome reconstitution included lactic acid bacteria and *Bifidobacterium* strains, which may affect the GI differently than *B. velezensis* DSM 33864 investigated here.

Interestingly, in both human fecal media and mouse fecal samples, *Bacillus* abundances were only enriched under conditions where clindamycin, *C. difficile* and *B. velezensis* DSM 33864 spores co-occurred, but not where *B. velezensis* DSM 33864 was administered alone to a non-antibiotic disrupted microbiome. While the underlying mechanism of this observation is unknown and warrants further investigation, the findings imply that *B. velezensis* DSM 33864 does not colonize a stable microbiome but requires antibiotic microbiota disruption to open a niche environment where it can then establish in the microbiota*.*

The exact mechanism by which *B. velezensis* DSM 33864 reduces *C. difficile* load in these studies is unclear, and likely involves multiple contributing factors. The strain has previously shown the ability to produce compounds capable of reducing *C. difficile* levels, which could contribute to the activity seen in this study^16^. Here, we also see *B. velezensis* DSM 33864 only establishing in the microbiota of mice that have been treated by antibiotics, similar to how *C. difficile* has the potential capacity to colonize the GI tract after antibiotic treatment has opened a niche in the microbiota^5^. These results lead us to suggest that *B. velezensis* DSM 33864 may outcompete *C. difficile* to occupy the environmental niche made available through antibiotic disruption of the microbiota. This competition dynamic could therefore lead to the exclusion of *C. difficile*, preventing its further proliferation in the GI tract.

Individuals harboring *C. difficile* and those who have previously developed CDI suffer from reduced quality of life, an often overlooked problem^22,23^. These individuals have a high risk of CDI recurrence and are often advised by health care providers to support their commensal microbiome as a means to reduce *C. difficile* load^23,24^. Our results indicate that *B. velezensis* DSM 33864 could act as a promising oral supplement for such individuals by reducing *C. difficile* colonization without impacting the commensal gut microbiota. This has led us to initiate a clinical trial in individuals who have previously experienced *C. difficile* infections where we will investigate the potential of *B. velezensis* DSM 33864 to reduce *C. difficile* colonization and to support healthy reconstitution of the gut microbiota composition and improve quality of life (Clinicaltrials.gov identifier NCT05606159).

## Materials and methods

### *B. velezensis* DSM 33864 spore preparation

*B. velezensis* DSM 33864 was previously classified as *B. amyloliquefaciens* and is also known as SG277. Sporulation of *B. velezensis* DSM 33864 was induced using Difco Sporulation broth (DSM). In brief, a single bacterial colony was transferred from Difco agar to 25 mL of Difco Sporulation broth and incubated 8 h at 37 °C with shaking at 150 rpm. After 8 h, 10 mL of culture was poured into a tray and incubated for 72 h at 37 °C. The bacterial lawn was harvested and resuspended in 10 mL of H_2_O. After washing and centrifugation (4.500 g, 10 min), spores were diluted to a concentration of 1 × 10^10^ spores/mL in ddH2O and stored at 4 °C until use.

### Human fecal fermentation assay and fluorescence in situ hybridization assay

Fecal fermentations were conducted under 5 different conditions in 4 replicates (Table 1). All fecal donors were healthy females between ages 18–65 years. The study was approved by the Clinical Research Ethics Committee of the Cork Teaching Hospitals (study number AFCRO-101) and conducted in accordance with the ICH Guidelines on Good Clinical Practice and the Declaration of Helsinki. Written informed consent was obtained from all participants and participants were free to withdraw from the study at any time. Samples have been tested negative for the following pathogens: Adenovirus, Astrovirus, Norovirus Genotype 1, Norovirus Genotype 2, Rotavirus, Sapovirus, Campylobacter spp., *C. difficile* toxins A/B, *Escherichia coli* 0157, *Salmonella* spp., *Shigella* spp./EIEC, STEC, *Yersinia enterocolitica* and Hepatitis A. Fecal matter from each donor was first normalized by mixing 10 g fecal material, 16 mL of 50% (w/v) glycerol and 14 mL water (cell grade) to a total of 40 mL fecal slurry. For the fermentations, a pool of fecal matter from four donors was used. Each fecal fermentation was composed of 1 mL fecal slurry spiked with the components specified in Table 1, made up to a final volume of 10 mL with pre-reduced BRM media^25^. In addition to the fecal slurry, regarding each condition composition, components were added in the following final concentrations: 0.45 mg/mL clindamycin (Sigma-Aldrich, Germany, CAS-No: 24729-96-2), 10^5^ CFUs/mL *C. difficile*, 10^8^ CFUs/mL of *B. velezensis* DSM 33864 spores. All fecal matter preparation and fermentations were conducted under strictly anaerobic conditions (0.0% O2, 10.0% CO_2_, 3% H_2_, in a Don Whitley M95 anaerobic workstation, UK). Fecal fermentations were incubated for 6 h at 37 °C and samples for *C.* *difficile* and *Bacillus* CFU quantification, microbiome profiling and FISH assays were collected at 0 h and 6 h of incubation. Material for microbiota compositional analysis was sampled from the fermentation tubes and centrifuged 5 min at 11,000 × g. The supernatant was aspirated and DNA was extracted from the remaining pellet following the protocol specified in the later section on 16S rRNA gene amplicon sequencing.

**Table 1:** Tested conditions for human fecal fermentation assay.

Group	Antibiotic	*C. difficile*	*B. velezensis* DSM 33864
Control	–	–	–
*B. velezensis* DSM 33864	–	–	10^8^ CFU/mL
Clindamycin	Clindamycin (0.45 mg/mL)	–	–
Clindamycin + *C. difficile*	Clindamycin (0.45 mg/mL)	10^5^ CFUs/mL	–
Clindamycin + *C. difficile* + *B. velezensis* DSM 33864	Clindamycin (0.45 mg/mL)	10^5^ CFUs/mL	10^8^ CFU/mL

*C. difficile* and *Bacillus* CFUs per mL were quantified in the fecal fermentations at the two time points by a serial dilution of the samples then streaked onto selective media either for *C. difficile* (Brazier’s Clostridium difficile Selective Medium, OXOID GmbH, Germany) or for *Bacillus* (Bacillus ChromoSelect Agar NutriSelect® Plus, Merck KGaA, Germany). The selective plates for *C. difficile* were incubated at 37 °C under strictly anaerobic conditions for 24–48 h. Selective plate media for the *Bacillus* genus were incubated at RT under aerobic conditions for 24–48 h. Identification of all colonies was confirmed by MALDI-TOF mass spectrometry (MALDI Biotyper®, Bruker) and CFU counts were adjusted to colonies identified only as either *C. difficile* or *Bacillus* genera. Calculation of CFUs/mL for each replicate and condition was performed considering the dilution factors.

Two groups of human fecal fermentations (Clindamycin + *C. difficile* ± *B. velezensis *DSM 33864) were selected for further analysis by FISH to visualize specific bacterial taxa. For this, 500 µL from each fecal fermentation were collected in duplicate and centrifuged at 4000 × g for 10 min with each duplicate fixated either as Gram-positive or -negative bacteria. For Gram-negative bacteria, the cell pellet was resuspended in 500 µL PBS 1X and 1.5 mL of 4% paraformaldehyde was added, homogenized, and incubated at 4 °C for 1 h. Cells were washed three times with PBS 1x. The cell pellet was resuspended with 250 µL PBS1x and 250 µL ethanol (99%), and samples were kept at − 20 °C. Fixation for Gram-positive bacteria differed in the first steps where cell pellets were resuspended in 500 µl of PBS1x and 500 µL of ethanol 98% and incubated at 4 °C for 4 h. FISH probes for *Bacillus* genus, *Clostridium* genus and higher taxonomy groups were chosen by searching either ProbeBase or peer-reviewed scientific literature^26^. The probes were synthesized with a fluorophore attached to its 5′ end, using Alexa Fluor or Cyanine chemistry and acquired from Eurofins Genomics, Germany. For probe details see Table 2. The protocol applied followed that described by Nielsen 2009^27^ with minor adaptations: no enzyme permeabilization was applied; the hybridization of *Bacillus* and *Clostridium* genera probes was conducted under 49 °C; Each taxa-specific probe was used in combination with a mix of universal bacterial probes (EUB338/EUB388II/EUB388III). FISH slides were observed under an Olympus FluoView FV3000 Confocal laser scanning Microscope, and images were analyzed using ImageJ software (v 1.52a, https://imagej.nih.gov/ij/index.html NIH, Bethesda, MD, USA). Semi-quantification of relative abundances was obtained by eye inspection of 20 fields for each replica of each sample analyzed by FISH, calculated as the percentage range of the target probe versus the *Eubacteria* probe.

**Table 2:** FISH probe information.

Target	Probe name	Fluorophore	Probe sequence	Formamide (%)	References
*Clostridium* (genus)	Cdiff193	Alexa488	TGTACTGGCTCACCTTTG	20	^28^
*Bacillus* (genus)	LGC353b	Alexa647	GCG GAA GAT TCC CTA CTG C	20	^29^
Firmicutes	LGC354A	Alexa488	TGG AAG ATT CCC TAC TGC	35	^30^
	LGC354B	Alexa488	CGG AAG ATT CCC TAC TGC		
	LGC354C	Alexa488	CCG AAG ATT CCC TAC TGC		
Eubacteria	EUB338I	Alexa488/Cy3/	GCT GCC TCC CGT AGG AGT	0–50	
	EUB338II	Alexa647	GCA GCC ACC CGT AGG TGT		
	EUB338III		GCT GCC ACC CGT AGG TGT		

### Mouse study of *C. difficile* colonization

To evaluate the efficacy of *B. velezensis* DSM 33864 on reduction of *C. difficile* colonization, a study of non-fatal and asymptomatic murine colonization model using 38 female C57BL/6 mice (Charles River) aged 7–8 weeks was carried out in accordance with the United Kingdom Animals (Scientific Procedures) Act 1986 and under a UK Home Office Project license PPL PP9830471. During the acclimatization and study mice were fed a non-restricted irradiated diet (5053, IPS), provided autoclaved tap water, and housed in cages containing wood fiber bedding (Lignocel), cardboard tunnels (LBS), Bed-r’Nests (Datesand) and chew blocks (LBS). After 31 days of acclimation mice were single caged (using HEPA-filtered independently ventilated cages) to avoid reinfection by coprophagia and were randomly allocated in 4 groups.

Mice were dosed by intragastric gavage (0.2 mL) with 30 mg/kg body weight of clindamycin (clindamycin hydrochloride C5269, Sigma, dissolved in 0.2 mL ddH_2_O) to deplete the commensal microbiota or 0.2 mL of water. After 5 h the mice were orally gavaged with 1 × 10^9^ spores of *B. velezensis* DSM 33864 in 0.2 mL PBS or an equal volume of PBS as the vehicle control. The gavage with *B. velezensis* DSM 33864 or vehicle was repeated 17 h after clindamycin gavage. *C. difficile* spores of strain 630 (TcdA^+^ TcdB^+^; Histodenz purified, prevalidated by CFU viability counting and for infectivity within mice) were diluted to a concentration of 1 × 10^3^ spores/mL in ddH2O using a screw cap glass serological test tube. The spore dose concentration was validated prior to challenge using viable counts on BHISS (37 g/L, 5 g yeast extract/L) agar containing 0.1% (w/v) sodium taurocholate. *C. difficile* (1 × 10^2^ spores in 0.1 mL) or an equal volume of water was dosed to mice by oral gavage 24 h after the clindamycin dose. 1 h following *C. difficile* administration the mice were gavaged with *B. velezensis* DSM 33864 or vehicle for a third time, which was repeated 6 h following *C. difficile* administration. The study was terminated 24 h after *C. difficile* administration, equivalent to 48 h after the single dose of clindamycin, as outlined in Fig S3a.

During the study, body weight, feed and water intake was monitored. Fecal pellets were collected at several time points (T1 (− 26 h), T2 (− 21 h), T3 (− 1 h) and T4 (22 h)) for microbiome analysis, while fecal samples from T4 were used for metabolome analysis. The mice were terminated by cervical dislocation followed by necropsy. Ceca were removed by dissection, stored on ice during collection, and then frozen at − 20 °C until subsequent analysis of *C. difficile* and toxin quantification. Murine cecum samples were centrifuged and pellets were suspended in 5 volumes of 80% (v/v) ethanol, vortexed vigorously for 5 m, and incubated for 1 h at RT before serial dilution and plating on selective media (CHROMID® *C. difficile*, bioMérieux, France). Plates were incubated for 24 h at 37 °C under anaerobic conditions. Colonies were counted and *C. difficile* CFU per g of sample calculated.

### Mouse study of gut microbiota composition impact

A mouse study to evaluate potential influence of *B. velezensis* DSM 33864 on the commensal and antibiotic-depleted gut microbiota composition was conducted using 60 female C57Bl/6Ncrl mice (Charles River). The mice were co-housed with three mice in each cage containing bedding (Tapvei), nesting material (Tapvei), wood brick (Tapvei), mouse igloo (Bio-Serv), Diamond Twist (Envigo), and a handling tube (Datesand). The mice were fed 1314 Fortified diet (Altromin) ad libitum and had non-restricted access to drinking water during the acclimatization period and the study. Following 18 days of acclimatization the mice were allocated randomly to 4 groups, including an arm of 2 groups receiving 30 mg/kg body weight of clindamycin and the remaining 2 groups receiving vehicle (PBS). In both the antibiotic and non-antibiotic arm each mouse received a daily dose of either *B. velezensis* DSM 33864 (1 × 10^6^ spores/dose) or PBS vehicle by oral gavage in a fixed volume of 0.2 mL. The mice received daily oral administration of the *B. velezensis* DSM 33864 or vehicle for 14 days, followed by a 14-day wash out period, as outlined in Fig S4a, e. At the start of the study, four hours prior to the first administration of *B. velezensis* DSM 33864 or vehicle, animals received either a single dose of clindamycin (30 mg/kg), or vehicle by oral gavage. Mice were weighed upon arrival and monitored weekly during the 4 weeks of the study. From the beginning of oral gavage, food consumption was recorded once weekly for each cage.

Fecal pellets were collected from all mice in the mornings prior to any oral gavage, a total of 6 timepoints, snap-frozen on dry ice and stored at − 80 °C. The first fecal sample was collected three days prior to the start of the study and prior to any oral gavage with clindamycin or *B. velezensis* DSM 33864. The second fecal sample was collected 24 h after the dose of clindamycin or vehicle and prior to the second *B. velezensis* DSM 33864 dose. From there a fecal sample was collected weekly. Fecal pellets were later used for microbiota composition analysis. Mice were euthanized by placement in a chamber with atmospheric air upon which a mixture of 70% CO_2_ and 30% O_2_ was applied with a steadily increasing concentration of CO_2_.

### Mouse study of *B. velezensis* DSM 33864 safety

To evaluate the safety of repeated dosing of *B. velezensis* DSM 33864 a study in both female and male mice with a 28-days repeat dose was conducted. A total of 54 C57Bl/6Ncrl mice (Charles River) (27 Male and 27 females) were used in the study. The study was performed in accordance with principles and considerations described in the OECD Guideline No. 407: Repeated Dose 28-Day Oral Toxicity Study in Rodents and in compliance with Principles of Good Laboratory Practice (GLP).

The mice were co-housed in cages with enrichment, feed and drinking water as described for the mouse study of gut microbiota composition. The mice were randomly allocated to 3 groups with 9 mice per sex in each. Following 19 days of acclimatization the mice received *B. velezensis* DSM 33864 in a dosage of 1 × 10^6^ or 1 × 10^8^ spores/day in 0.2 mL PBS by oral gavage or equal volume PBS for 28-days, as outlined in Fig S4b. During the 28-day treatment period, clinical signs were monitored daily, body weight and food consumption recorded weekly. Mice were euthanized by placement in a chamber with atmospheric air upon which a mixture of 70% CO_2_ and 30% 0_2_ was applied with a steadily increasing concentration of CO_2_. Death was confirmed and the animals were bled before proceeding with necropsy. At termination, blood samples were collected by retro-orbital bleeding. Samples for hematology and coagulation parameters were collected from 5 males to 4 females from each group. For hematology, at least 150 µL K_3_ EDTA stabilized blood was taken and samples were diluted 1:1 with ABX Diluent before analysis and the results were corrected where appropriate. For coagulation tests, 250 µL citrate stabilized blood was taken. At least 500 µL blood was taken for clinical chemistry in tubes with clotting activator for serum. A macroscopic examination was performed after opening the cranial, thoracic, and abdominal cavities by observing the appearance of the organs and tissues in situ. Any macroscopic change was recorded with details of the location, color, shape and/or size in Provantis version 9.3.0.0 (Instem). All tissues were fixed in phosphate buffered neutral 4% (v/v) formaldehyde except for the eyes and testes (Modified Davidson’s fixative). The lungs were infused with fixative at necropsy.

### *C. difficile t*oxin quantification by ELISA

TcdA and TcdB were quantified from cecum content from the murine *C. difficile* colonization study. The wet weight of material was determined, and samples were suspended in 5 volumes of toxin extraction buffer (50 mL of buffer contains 1 mL Fetal Bovine Serum, 2 Pierce Protease Inhibitor tablets, 100 μL PMSF, 50 μL Benzamide, 500 μL 100 × Penicillin–Streptomycin, 500 μL EDTA and 47.85 mL PBS). Samples were then macerated, and toxins extracted for 2 h at 4 °C with gentle agitation. Tubes were centrifuged (microfuge, 4 °C, 15 min at 10,000 rpm) and the supernatant filtered through a 0.2 μm Corning syringe filter. Filtered supernatant was stored at 4 °C prior to use. Samples were analyzed for the presence of TcdA and TcdB by ELISA (tcgBIOMICS) and the amount of toxins per g of sample determined by following the manufacturer’s instructions.

### Microbiota composition analysis by 16S rRNA gene amplicon sequencing

DNA was extracted using NucleoSpin® 96 Soil (Macherey–Nagel) by adding lysis buffer directly to human fecal fermentation or mouse fecal pellets in sterile tubes and this mix was transferred to the beads tube provided in the extraction kit. Amplification of the 16S RNA gene by PCR from human fecal media and mouse fecal pellets from the microbiome safety study was done with primers targeting the V3–V4 region (341.2FDI and 805.2RDI). The following PCR program was used: 98 °C for 2 min, 9x (98 °C for 30 s, 52 °C for 30 s, 72 °C for 30 s), 72 °C for 5 min followed by a hold at 4 °C. Indexes were added in a subsequent PCR with the following PCR program: 98 °C for 2 min, 9x (98 °C for 30 s, 55 °C for 30 s, 72 °C for 30 s), 72 °C for 5 min and a hold at hold at 4 °C.

The PCR product was purified using AMPure beads (Beckman Coulter) and samples were pooled in equimolar amounts based on DNA concentrations determined fluorometrically. Sequencing was done on an Illumina MiSeq desktop sequencer using the MiSeq Reagent Kit V3 (Illumina) for 2 × 300 bp paired-end sequencing. Sequence data was processed using dada2^31^. Primer sequences were first removed from raw reads (dada2::p-trim-left-f/r, 19 bases from the forward read and 20 from the reverse read). Reads were then trimmed at the 3’ ends based on quality scores, but keeping a minimum read lengths of 279 and 209 bp for forward and reverse reads, respectively (dada2::filterAndTrim). All trimmed reads with more than two expected errors were discarded. The remaining reads were dereplicated into unique sequences and then denoised separately for forward and reverse reads. Denoised forward and reverse reads were then merged, thereby discarding read pairs without sufficient overlap (12 bp) or with any mismatch in the overlap. Finally, suspected chimeras were removed from the generated abundance table by internal abundance and sequence comparisons. The default taxonomic assignment of the detected amplicon sequence variants (ASVs) was done using a custom naïve Bayesian classifier algorithm comparing the ASV sequences to the SILVA reference database^32^ (v138). Singleton ASVs were removed from downstream analyses.

Amplification of the 16S RNA gene by PCR from the mouse colonization study was carried out using primers of the V3-V4 region (S-D-Bact-0341-b-S-17 and S-D-Bact-0785-a-A-21)^33^. Indexes were added in a subsequent PCR using the Nextera Index Kit V2 (Illumina) and PCR products were pooled based on concentration and resulting library cleaned with AMPure beads (Beckman Coulter). Sequencing was done on an Illumina MiSeq desktop sequencer using the MiSeq Reagents Kit V3 (Illumina) for 2 × 300 bp paired-end sequencing. An adjusted dada2 pipeline was used for bioinformatics processing of the sequence data into the ASV (amplicon sequence variant) abundance table^31^. In the first step, primer sequences were removed from raw reads using cutadapt^34^. Reads without perfect primer match or with ambiguous bases, as well as reads shorter than 275 bp, were filtered out. In an additional filtering and trimming step (dada2::filterAndTrim command), reads were first trimmed at the 3 prime ends based on quality scores, but keeping a minimum read length of 250 and 220 bp for forward and reverse reads, respectively. All trimmed reads with more than 1 expected error were discarded. The remaining reads were dereplicated into unique sequences and then denoised separately for forward and reverse reads. Denoised forward and reverse reads were then merged, thereby discarding read pairs without sufficient overlap (20 bp) or with any mismatch in the overlap. Finally, suspected chimeras were removed from the generated abundance table by internal abundance and sequence comparisons. The default taxonomic assignment of the detected ASVs was done using a naïve Bayesian classifier algorithm comparing the ASV sequences to the SILVA reference database (v138). For improving the taxonomic assignment of the ASVs, we in-silico extracted amplicons corresponding to the primers from current versions of four reference databases (SILVA^32^, RDP^35^ and UHGG^36^). For each ASV, we used reference amplicons matching perfectly to the ASV, or the amplicons with the highest sequence identity to the ASV, to check/improve the default assignment of the ASV.

All ASV count tables generated from human fecal media and both mouse studies were analyzed using R^37^. Zero imputation was performed using zCompositions, and counts were subsequently center-log transformed using compositions^38,39^. Ordinations were performed using Principal Component Analysis (PCA) on the subsequent pseudo-counts^40^. Alpha diversity was calculated according to the Shannon diversity index^41^. All tests for differences between groups were performed using permutational multiple analysis of variance (perMANOVA) with Euclidean distance matrices, where differences were considered significant if *p*-values were less than 0.05^42,43^. Differences in abundance of ASVs between groups identified as significantly different by perMANOVA were computed using Tukey’s Honest Significant Differences test^44^.

### Cyclic lipopeptide quantification by metabolomics analysis of mouse fecal samples LCMS

Fecal samples were extracted with MeOH (75%) from the mouse study of *C. difficile* colonization. Extracts were mixed for 60 min followed by centrifugation (5000 g for 5 min). The supernatants were transferred to glass vials and 15 μL was injected (on a 10 μL loop) onto a reversed-phase chromatography and mass spectrometer (LCMS). The LC-system was an Accela, and the MS was a Q Exactive™ Hybrid Quadrupole-Orbitrap Mass Spectrometer both from Thermo Fisher Scientific, Denmark. The MS was operated in positive and negative ion mode electrospray ionization (ESI +) and (ESI−). Data were collected from 123 to 1500 m/z (enabling detection of molecules with a molecular weight of 123 Da to more than 10.000 Da) The chromatographic part of the LCMS-system was setup with a CSH-peptide C18 column (1.7 μm particle, 150 mm, 2.1 mm ID, Waters) using a gradient system with Eluent A: water with 0.1% formic acid, Eluent B: Acetonitrile with 0.1% formic acid and Eluent C: Isopropanol with 0.1% formic acid. A flow of 0.250 mL/min was used, starting at 99.5% A-eluent lowering to 50% and increasing with 50% B-eluent within 17 min, followed by a new gradient to endpoint 20:40:40 eluent A: B:C at 22 min. The gradient was kept isocratic until 24 min and the eluent returned to initial conditions at 26 min. All gradients were linear.

Peaks were extracted by the MS-data processing program Refiner Expressionist 16.0 by Genedata Data Analysis Systems software (Genedata). For identification a database with retention time and accurate mass has been used. The workflow was built form various modules (nodes). First, noise subtraction was performed by smoothing chromatograms over 5 MS-scans and noise over 51 scans and then removing the 65% quantile signal. Retention time (RT)-alignment was done via Pairwise Alignment Bases Tree, a parameter within the retention time alignment activity, that corrects retention time shifts enabling better comparison of chromatograms and facilitating peak and compound detection. Peak detection was Ascent-based and done in 10 scan windows, where peak-splitting was allowed. Isotope clustering was done with 0.1 min RT and 7 ppm mass tolerance, allowing charges from 1 to 12 and 1 to 5 for ESI + and ESI− collection, respectively. Adduct grouping was based on H + , Na + , H2O-neutal loss for ESI + collection and H–, HCOOH– and H_2_O– neutal loss for ESI- collection. Identification was based on a proprietary, custom database with more than 100,000 molecules. For data analysis, metabolites were normalized by sample weight, scaled, and centered per sample. Targeted metabolites were scaled and centered to compare metabolite abundances between cyclic lipopeptides.

### Statistical analysis

16S data were analyzed as described in the designated section. All other measures were visualized and analyzed statistically as described in figure legends using GraphPad Prism 9. Longitudinal measures were analyzed by two-way ANOVA with Dunnett’s multiple comparisons test while single timepoint measures were measured one-way ANOVA with Tukey’s multiple comparisons test. Asterisks in graphs illustrate corrected *p* < 0.05 between indicated groups where *< 0.05, **< 0.01 and ***< 0.001.

### Supplementary Information

Supplementary Figure S1.

Supplementary Figure S2.

Supplementary Figure S3.

Supplementary Figure S4.

## Data Availability

The raw data can be made available upon request to the corresponding author.

## References

[1] Czepiel J (2019). *Clostridium difficile* infection. Eur. J. Clin. Microbiol. Infect. Dis..

[2] Schaeffler H, Breitrueck A (2018). *Clostridium difficile*–from colonization to infection. Front. Microbiol..

[3] Girinathan BP (2021). In vivo commensal control of *Clostridioides difficile* virulence. Cell Host Microbe.

[4] Sorg JA, Sonenshein AL (2008). Bile salts and glycine as cogerminants for *Clostridium difficile* spores. J. Bacteriol..

[5] Chang JY (2008). Decreased diversity of the fecal microbiome in recurrent *Clostridium difficile*—associated diarrhea. J. Infect. Dis..

[6] Jenior ML, Leslie JL, Young VB, Schloss PD (2017). *Clostridium difficile* colonizes alternative nutrient niches during infection across distinct murine gut microbiomes. MSystems.

[7] Smith AB (2022). Enterococci enhance *Clostridioides difficile* pathogenesis. Nature.

[8] Fletcher JR (2021). *Clostridioides difficile* exploits toxin-mediated inflammation to alter the host nutritional landscape and exclude competitors from the gut microbiota. Nat. Commun..

[9] Darkoh C, DuPont HL, Norris SJ, Kaplan HB (2015). Toxin synthesis by *Clostridium difficile* is regulated through quorum signaling. MBio.

[10] Di Bella S, Ascenzi P, Siarakas S, Petrosillo N, Di Masi A (2016). *Clostridium difficile* toxins A and B: Insights into pathogenic properties and extraintestinal effects. Toxins.

[11] Smits WK, Lyras D, Lacy DB, Wilcox MH, Kuijper EJ (2016). *Clostridium difficile* infection. Nat. Rev. Dis. Primers..

[12] Dawkins JJ (2022). Gut metabolites predict *Clostridioides difficile* recurrence. Microbiome.

[13] Khoruts A, Staley C, Sadowsky MJ (2021). Faecal microbiota transplantation for Clostridioides difficile: mechanisms and pharmacology. Nat. Rev. Gastroenterol. Hepatol..

[14] Baunwall SMD (2020). Faecal microbiota transplantation for recurrent Clostridioides difficile infection: An updated systematic review and meta-analysis. EClinicalMedicine.

[15] Goldenberg JZ (2017). Probiotics for the prevention of *Clostridium difficile*-associated diarrhea in adults and children. Cochrane Database Syst. Rev..

[16] Ferreira WT (2021). Micellar antibiotics of *Bacillus*. Pharmaceutics.

[17] Cho MS, Jin YJ, Kang BK, Park YK, Kim C, Park DS (2018). Understanding the ontogeny and succession of *Bacillus velezensis* and *B. subtilis* subsp. subtilis by focusing on kimchi fermentation. Sci Rep.

[18] Hazards EPOB (2023). Update of the list of qualified presumption of safety (QPS) recommended microbiological agents intentionally added to food or feed as notified to EFSA 17: Suitability of taxonomic units notified to EFSA until September 2022. EFSA J..

[19] Lee NK, Kim WS, Paik HD (2019). Strains as human probiotics: characterization, safety, microbiome, and probiotic carrier. Food Sci. Biotechnol..

[20] Suez J (2018). Post-antibiotic gut mucosal microbiome reconstitution is impaired by probiotics and improved by autologous FMT. Cell.

[21] O’Donnell MM (2022). Identification of ADS024, a newly characterized strain of *Bacillus* velezensis with direct *Clostridiodes difficile* killing and toxin degradation bio-activities. Sci. Rep..

[22] Han Z, Lapin B, Garey KW, Donskey CJ, Deshpande A (2022). Impact of Clostridioides difficile infection on patient-reported quality of life. Infect. Control Hosp. Epidemiol..

[23] Stephenson AL (2022). Seeing C diff differently. Lancet Gastroenterol. Hepatol..

[24] Goldenberg JZ, Mertz D, Johnston BC (2018). Probiotics to prevent *Clostridium difficile* infection in patients receiving antibiotics. JAMA.

[25] Robinson CD, Auchtung JM, Collins J, Britton RA (2014). Epidemic *Clostridium difficile* strains demonstrate increased competitive fitness compared to nonepidemic isolates. Infect. Immun..

[26] Greuter D, Loy A, Horn M, Rattei T (2016). probeBase—an online resource for rRNA-targeted oligonucleotide probes and primers: New features 2016. Nucl. Acids Res..

[27] Nielsen P, Daims H (2009). FISH Handbook for Biological Wastewater Treatment.

[28] Smith B (2011). Community analysis of bacteria colonizing intestinal tissue of neonates with necrotizing enterocolitis. BMC Microbiol..

[29] Felske A, Akkermans AD, De Vos WM (1998). In situ detection of an uncultured predominant *Bacillus* in Dutch grassland soils. Appl. Environ. Microbiol..

[30] Meier H, Amann R, Ludwig W, Schleifer KH (1999). Specific oligonucleotide probes for in situ detection of a major group of gram-positive bacteria with low DNA G+ C content. Syst. Appl. Microbiol..

[31] Callahan BJ, McMurdie PJ, Rosen MJ, Han AW, Johnson AJ, Holmes SP (2016). DADA2: High-resolution sample inference from Illumina amplicon data. Nat. Methods.

[32] Quast C (2013). The SILVA ribosomal RNA gene database project: improved data processing and web-based tools. Nucl. Acids Res..

[33] Klindworth A (2013). Evaluation of general 16S ribosomal RNA gene PCR primers for classical and next-generation sequencing-based diversity studies. Nucl. Acids Res..

[34] Kechin A, Boyarskikh U, Kel A, Filipenko M (2017). cutPrimers: A new tool for accurate cutting of primers from reads of targeted next generation sequencing. J. Comput. Biol..

[35] Cole JR (2014). Ribosomal Database Project: data and tools for high throughput rRNA analysis. Nucl. Acids Res..

[36] Mitchell AL (2020). MGnify: the microbiome analysis resource in 2020. Nucl. Acids Res..

[37] R. C. Team (2021). R: a language and environment for statistical computing. Vienna: R Foundation for Statistical Computing.

[38] Palarea-Albaladejo J, Martín-Fernández JA (2015). zCompositions—R package for multivariate imputation of left-censored data under a compositional approach. Chemom. Intell. Lab. Syst..

[39] Van den Boogaart KG, Tolosana-Delgado R (2008). “Compositions”: a unified R package to analyze compositional data. Comput. Geosci..

[40] McGarigal K, Cushman SA, Stafford S (2013). Multivariate statistics for wildlife and ecology research.

[41] Shannon CE (1948). A mathematical theory of communication. The Bell system technical journal.

[42] Warton DI, Wright ST, Wang Y (2012). Distance-based multivariate analyses confound location and dispersion effects. Methods Ecol. Evol..

[43] Oksanen, J., Blanchet, F.G., Kindt, R., Legendre, P., Minchin, P.R., O’hara, R.B., Simpson, G.L., Solymos, P., Stevens, M.H.H. & Wagner, H., Vegan: Community ecology package. (R package version 2.0–2, 2012)

[44] Yandell BS (2017). Practical data analysis for designed experiments.

