# Supplementary material for: Bacillus velezensis DSM 33864 reduces Clostridioides difficile colonization without disturbing commensal gut microbiota composition
Source: Sci Rep. 2023 Sep 11;13:14941. doi: 10.1038/s41598-023-42128-8 (PMC10495459; doi:10.1038/s41598-023-42128-8)
Supplement: Supplementary file 1 — Supplementary Figure S1. [file 41598_2023_42128_MOESM1_ESM.pdf]

Figure S1

a

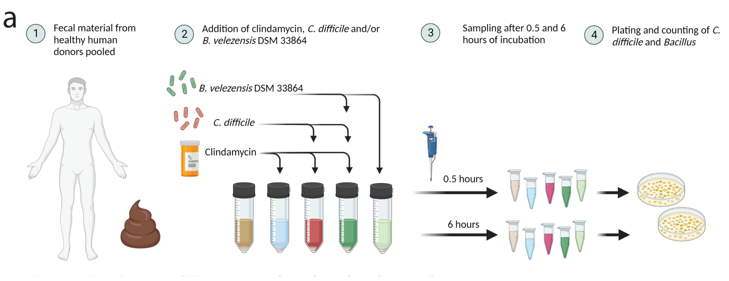

b

Target relative abundance (6h)	Clindamycin + *C. difficile*	Clindamycin + *C. difficile + B. velezensis DSM 33864*
Firmicutes	65-80 %	> 80 %
Clostridia	6-20 %	6-20 %
Bacillus	Not quantifiable	0.6-5 %

c

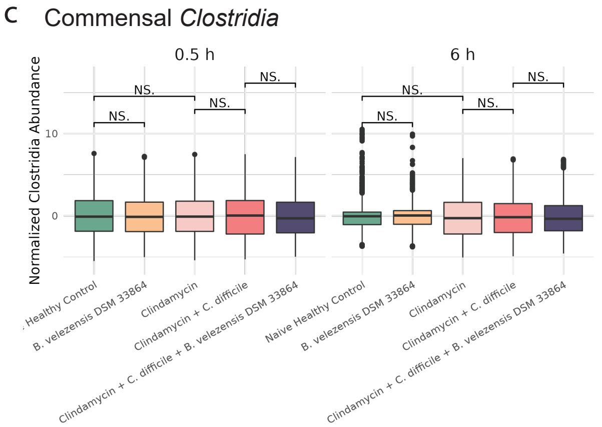

d

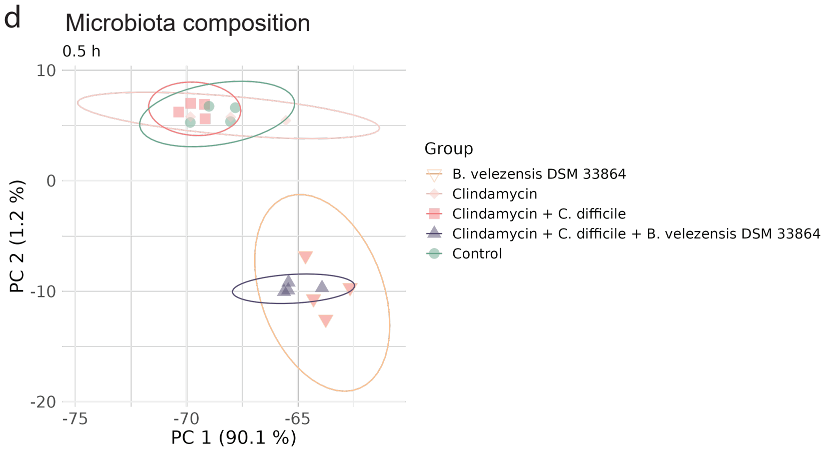

e

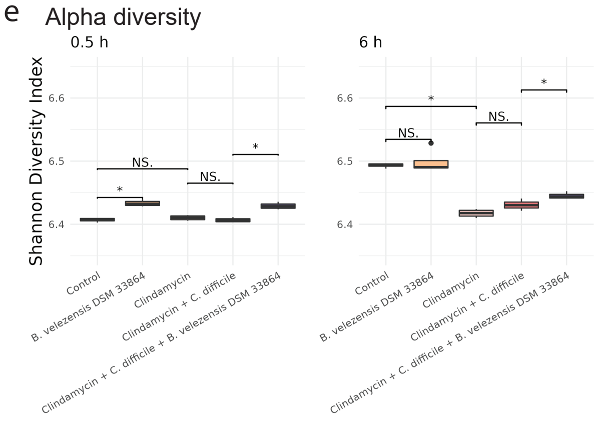

a) Experimental setup of human fecal incubations where pools of fecal samples from healthy donors were incubated with/without clindamycin, *C. difficile* or *B. velezensis* DSM 33864 as indicated with samples in 4 replicas sampled after 0.5 and 6 h of incubation. Figure created with BioRender.com. b) Relative semi-quantitative abundance by FISH of Firmicutes, Clostridia and Bacilli in human fecal incubations for 6 h with clindamycin and *C. difficile* with/without *B. velezensis* DSM 33864. c) Centered log-ratio (Clr) transformed commensal *Clostridia* abundance by 16S rRNA gene amplicon sequences aggregated at phylogenetic class level in human fecal incubations after 0.5 h and 6 h. d) Principal component analysis of 16S rRNA gene amplicon sequences of human fecal incubation 0.5 h with/without clindamycin, *C. difficile* or *B. velezensis* DSM 33864. e) Shannon diversity index in human fecal incubations after 0.5 h and 6 h with/without clindamycin, *C. difficile* or *B. velezensis* DSM 33864. c,e) Asterisks indicate p-values < 0.05 and ns indicate p-values > 0.05 comparing the groups by t-test.
