# Supplementary material for: Bacillus velezensis DSM 33864 reduces Clostridioides difficile colonization without disturbing commensal gut microbiota composition
Source: Sci Rep. 2023 Sep 11;13:14941. doi: 10.1038/s41598-023-42128-8 (PMC10495459; doi:10.1038/s41598-023-42128-8)
Supplement: Supplementary file 2 — Supplementary Figure S2. [file 41598_2023_42128_MOESM2_ESM.pdf]

Figure S2

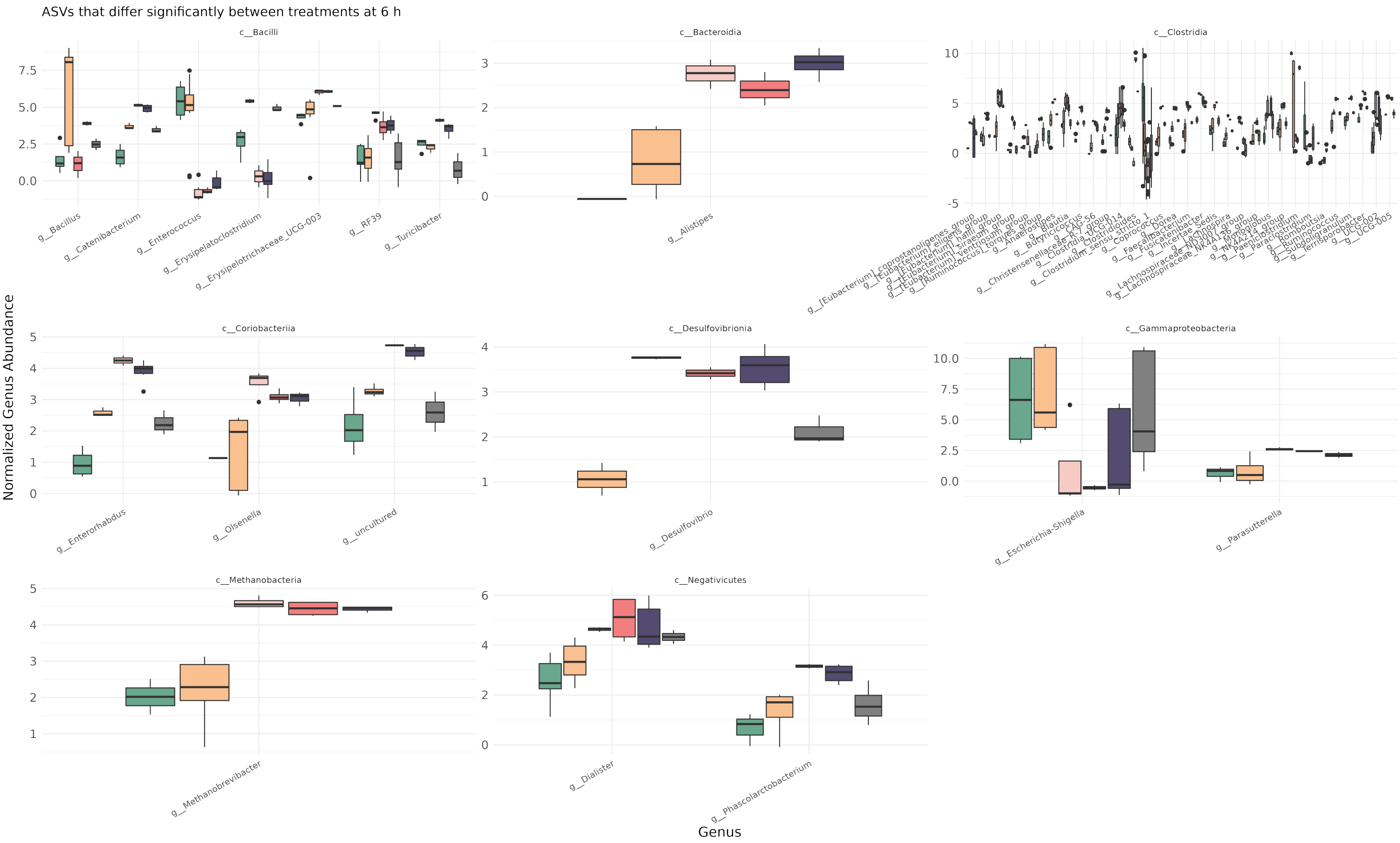

Clr transformed amplicon sequence variants statistically significantly changed between the groups in relative abundance in human fecal incubations after 6 h
