# Supplementary material for: Bacillus velezensis DSM 33864 reduces Clostridioides difficile colonization without disturbing commensal gut microbiota composition
Source: Sci Rep. 2023 Sep 11;13:14941. doi: 10.1038/s41598-023-42128-8 (PMC10495459; doi:10.1038/s41598-023-42128-8)
Supplement: Supplementary file 3 — Supplementary Figure S3. [file 41598_2023_42128_MOESM3_ESM.pdf]

Figure S3

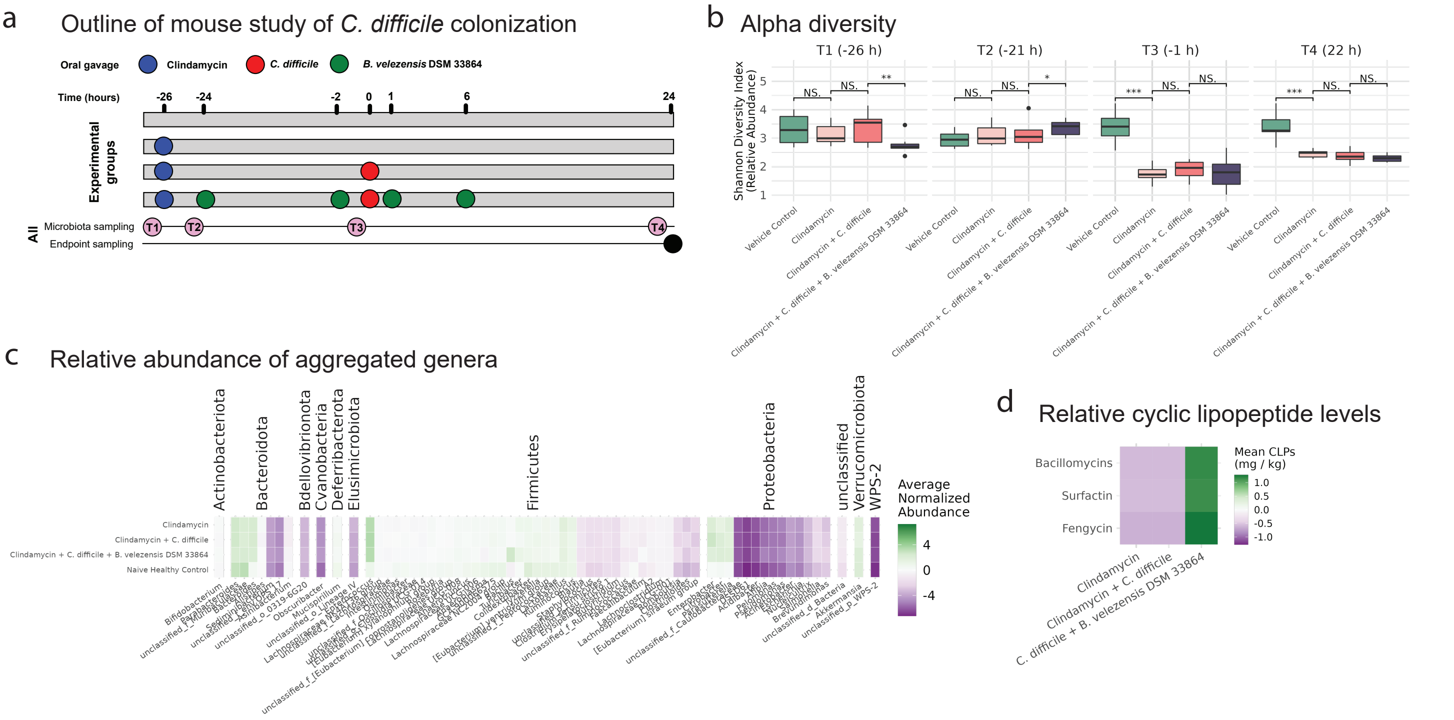

a) Experimental outline of *C. difficile* colonization study in mice given clindamycin or vehicle followed by a total of four oral gavages of *B. velezensis* DSM 33864 spores before and after oral gavage of *C. difficile*. During the study, fecal samples were collected at baseline prior any treatments (T1), after clindamycin-administration (T2), after the first two oral vehicle/*B. velezensis* DSM 33864 administrations (T3), and at the end of the study 24 hours after oral vehicle/*C. difficile* administration (T4). b) Shannon diversity index per group assessed by 16S rRNA gene amplicon sequencing of mouse fecal samples timepoints T1-4. Asterisks indicate p-values < 0.05 and ns indicate p-values > 0.05 comparing the groups by t-test. c) Heatmap of aggregated genus abundances of mouse fecal samples 24 hours after oral administration of *C. difficile* in the groups. d) Average relative levels of cyclic lipopeptides in mouse cecum per group sampled at 24 h after oral administration of *C. difficile* (T4) in indicated groups and measured by targeted metabolomics.
