# Supplementary figures and images for: Bacillus velezensis DSM 33864 reduces Clostridioides difficile colonization without disturbing commensal gut microbiota composition
Source: Sci Rep. 2023 Sep 11;13:14941. doi: 10.1038/s41598-023-42128-8 (PMC10495459; doi:10.1038/s41598-023-42128-8)

Figure S4

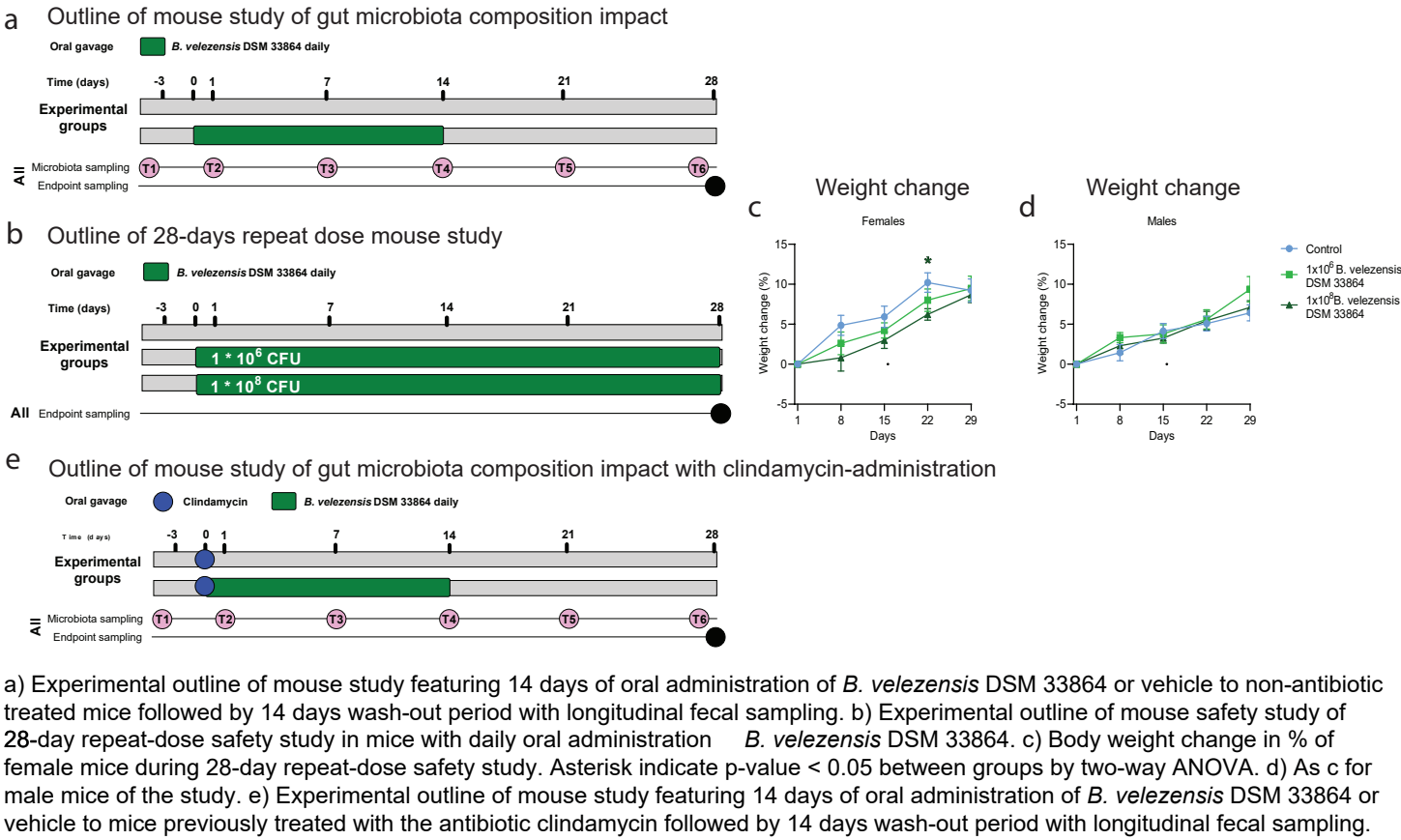

Supplement: Supplementary file 4 — Supplementary Figure S4. [file 41598_2023_42128_MOESM4_ESM.pdf]
